# SiC Heterojunction Trench MOSFET with a Buried P-Type Pillar for the Low Gate-Drain Charge and Switching Loss

**DOI:** 10.3390/mi13020248

**Published:** 2022-02-01

**Authors:** Shenglong Ran, Zhiyong Huang, Shengdong Hu, Han Yang

**Affiliations:** Chongqing Engineering Laboratory of High Performance Integrated Circuits, School of Microelectronics and Communication Engineering, Chongqing University, Chongqing 400044, China; rsl13983760794@163.com (S.R.); hushengdong@hotmail.com (S.H.); z15730536211@163.com (H.Y.)

**Keywords:** SiC MOSFET, assistant depletion, switching loss, gate-drain charge

## Abstract

A novel Silicon-Carbide heterojunction U-MOSFET embedded a P-type pillar buried in the drift layer (BP-TMOS) is proposed and simulated in this study. When functioning in the on state, the merged heterojunction structure will control the parasitic body diode, and the switching loss will decrease. Moreover, to lighten the electric field on the gate oxide corner, a high-doped L-shaped P^+^ layer near the heterojunction beneath the gate oxide was introduced; thus, the gate oxide reliability improved. A p-type pillar is introduced in the drift layer. The p-type pillar can assistant the drift layer to deplete. Thus, the specific on-resistance for BP-TMOS can be reduced with an increase in the N-drift region’s doping concentration. Compared to the traditional SiC MOSFET (C-TMOS), the specific on-resistance decreased by 20.4%, and the breakdown voltage increased by 53.7% for BP-TMOS, respectively. Meanwhile the device exhibits a 55% decrease and a 69.7% decrease for the switching loss and gate to drain charge.

## 1. Introduction

Over the years, the wide band-gap material has become a new research topic in the power devices. Silicon–carbide devices is a major type used in the power systems. The low on-state resistance, higher power density, increased high work frequency, and the widely forbidden band all stand out [1,2,3]. The small size and high reliability of the SiC MOSFET are both necessary to minish power dissipation and increase the efficiency. To avoid the aged deterioration effect, when the SiC MOSFET is used in the power system, usually paralleled with a Schottky barrier diode (SBD) [4,5,6,7], it may cause more energy dissipation and undesirable stray inductances. In order to obtain the optimised the reverse recovery performance and reduce switching loss, several papers have proposed several structures, for example, using a SBD paralleled with the SiC trench MOSFET [8,9,10,11,12]. In addition, in the trench SiC MOSFET, the gate oxide corner reliability is also an issue of interest. Under the trench gate, surrounding a highly doped P+ layer is an effective method.

Compared to the SiC SBD, two types of poly-silicon/SiC formed the heterojunction diode, which have better performance on the third quadrant. In this paper, the novel structure that buried a P-type pillar in drift layer has been researched. In addition to the optimised reverse recovery performance and the reduced the switching loss from the Heterojunction diode (HJD) in the drift region, the assist depletion effect from the P-pillar can bring a better balance among the specific on-resistance (*R*_on,sp_) and the breakdown voltage (*BV*). The dynamic and static performances of the proposed structure have been carefully investigated.

## 2. Device Structure and Mechanism

**Figure 1:**
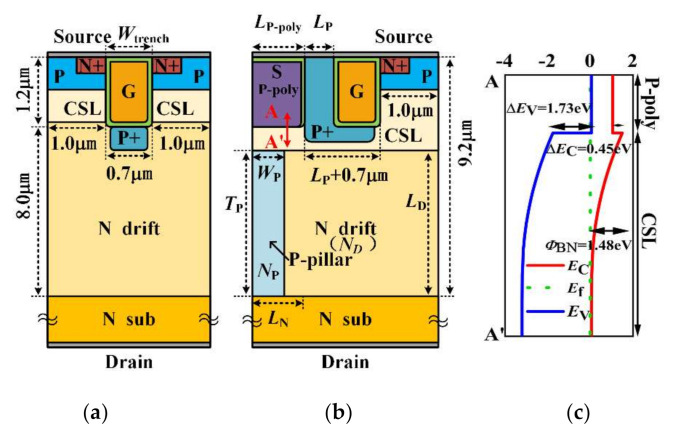
(**a**) C-TMOS; (**b**) BP-TMOS; (**c**) the energy band among the poly-silicon/SiC.

Figure 1a,b show the structures of the BP-TMOS that buried a P-type pillar and the traditional SiC MOSFET(C-TMOS). The energy band along the poly-silicon/SiC in the BP-TMOS is also shown in Figure 1c. In comaprison to the traditional one, BP-TMOS also has a heterojuction structure, which is composed of the poly-silicon/SiC. The heterojuction structure has a conduction band of 0.45 eV and valence band of 1.73 eV, respectively. An electron barrier height *Φ*_BN_ (about 1.48 eV) is caused by conduction band *E*_C_ and the Fermi-band *E*_f_. When the device is in a forward state, the height of this barrier will result in a smaller forward voltage (*V*_F_). Conversely, in a reverse position, the breakdown voltage *BV* is enhanced. Beneath the gate oxide and near the heterojunction, using a highly doped L-shaped P+ region reduced the gate oxide maximum electric field and improved the gate oxide layer’s reliability [13,14,15]. In addition, in the drift region near the gate oxide corner, the electric field concentration can lower by the L-shaped P+ layer and narrow the overlap area between the drift layer and the gate oxide layer and, thus, can achieve a fast-switching time and a high *BV*. From the work mechanism, the P-type pillar will afford an assistant depletion effect. Thus, the lateral P-pillar/N-drift junction and the vertical P-base/N-drift junction consist of the total depletion in drift layer. The drift layer doping concentration can be increased. Therefore, *R*_on,sp_ can be reduced and *BV* can be increased at the same time.

For practice, the device was placed in a tape-out process at the interface of SiO_2_, and SiC introduced the traps with a uniform concentration 5 × 10^12^ eV^−1^ cm^−2^. Table 1 lists the basic parameters of the two devices. In this paper, the devices were analysed by the Sentaurus TCAD tool. In a recombination process, Auger recombination, Shockley–Read–Hall (SRH), and doping-dependent methods are used, and Okuto–Crowell models are used in the electron/hole continuity equations [16]. The mobility model used Enormal, high-field velocity saturation, and incomplete ionization.

## 3. Results and Discussions

**Figure 2:**
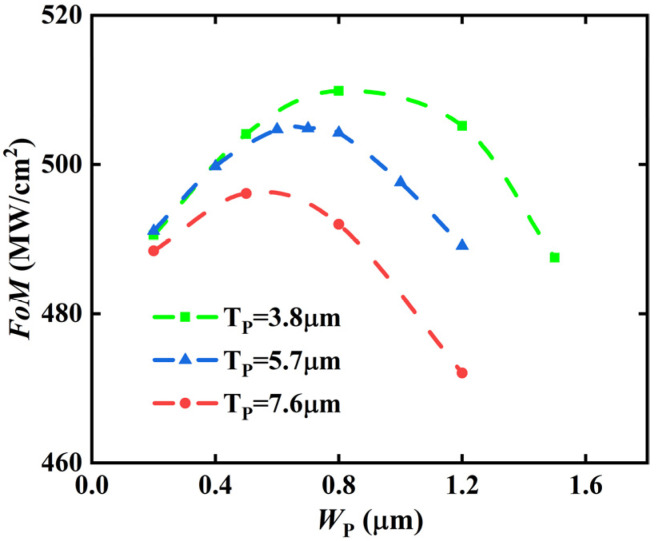
The *FoM* with different *W*_P_ and *T*_P_ for the BP-TMOS.

Figure 2 shows the *FoM* (Figure of Merit, *BV*^2^/*R*_on_) with different *W*_P_ and *T*_P_ for the BP-TMOS. Fixing a certain *T*_P_ of 3.8 μm, 5.7 μm, or 7.6 μm, *FoM* increases at first and then declines with an increase in *W*_P_. There is a maximum point for the *FoM*. The *T*_P_ is chosen when it is equal to the drift thickness (*L*_D_), and *W*_P_ is chosen when the largest *FoM* is obtained. Just as shown in Figure 2, the optimised values of *W*_P_ and *T*_P_ are 0.5 μm and 7.6 μm for BP-TMOS, respectively.

**Figure 3:**
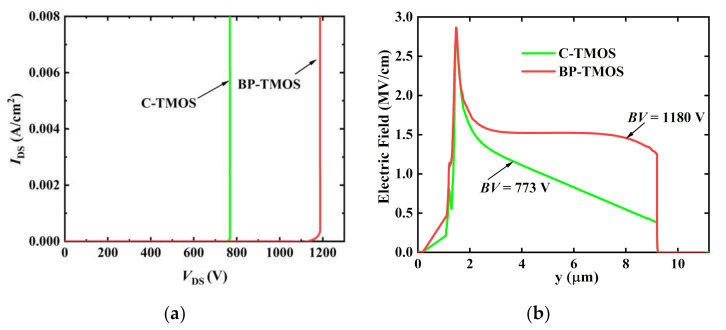
(**a**) The off-state *I*-*V* curves; (**b**) the electric field distribution sat breakdown.

Figure 3 provides the off-state characteristics about the proposed one and the traditional one. Figure 3a includes the off-state *I*-*V* curves. It clearly shows that the *BV*s of BP-TMOS and C-TMOS are 1180 V and 773 V, respectively. The total drain-source current *I*_ds_ achieves 10 μA/cm^2^ when considering its breakdown. Figure 3b shows the electric field distributions of BP-TMOS and C-TMOS on the vertical direction when the breakdown takes place. The electric field distribution in drift layer of BP-TMOS is uniform due to the assistant depletion effect of the P-pillar and wider P+ layer; thus, it has a higher *BV*.

**Figure 4:**
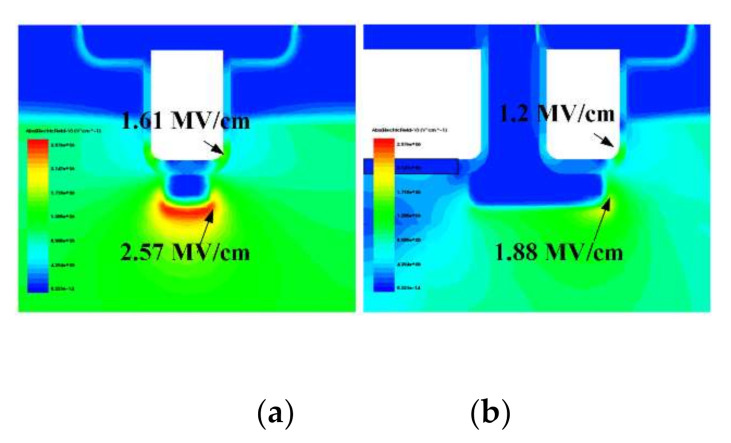
(**a**) Electric field distributions in C-TMOS; (**b**) electric field distributions in BP-TMOS.

Figure 4 provides the 2D electric field graphics of BP-TMOS and C-TMOS in the same drain voltage of 650 V. For BP-TMOS, the gate oxide corner maximum electric field (*E*_ox_) is 1.2 MV/cm, which is lower than 1.45 MV/cm for the C-TMOS. That is to say that the gate oxide reliability of BP-TMOS is better. In addition, the SiC region maximum electric field (*E*_SiC_) in BP-TMOS is also much lower, which means the premature breakdown near the P+ shielding layer is restrained for BP-TMOS, and a higher BV is, therefore, obtained as shown in Figure 3.

**Figure 5:**
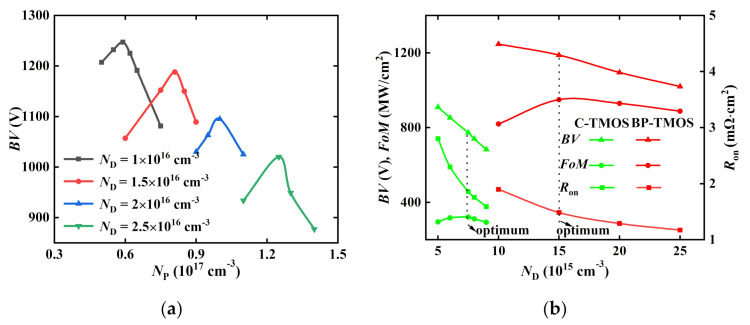
(**a**) The dependences *N*_D_ and *N*_P_ in *BV* for the BP-TMOS; (**b**) the optimised values between *R*_on,sp_ and *BV* for the BP-TMOS and the C-TMOS.

Figure 5 provides the devices’ electric performances with different structure parameters. Figure 5a shows the dependences on *BV* with different *N*_P_ and *N*_D_ for the BP-TMOS. It can be observed that there is a maximum point of *BV* for certain *N_D_*. Due to mutual depletion, a higher *N_D_* needs a higher *N_P_* to obtain the maximum *BV*. Figure 5b provides the optimizations of *BV* and *R*_on,sp_ for BP-TMOS and C-TMOS. The criteria conditions include *FoM*. The optimised value of *N*_D_ for BP-TMOS is chosen as 1.5 × 10^16^ cm^−3^, while in C-TMOS, it is 7.5 × 10^15^ cm^−3^, and an *N*_P_ of 8.1 × 10^16^ cm^−3^ was chosen. Compared to C-TMOS, BP-TMOS has a larger *FoM*.

**Figure 6:**
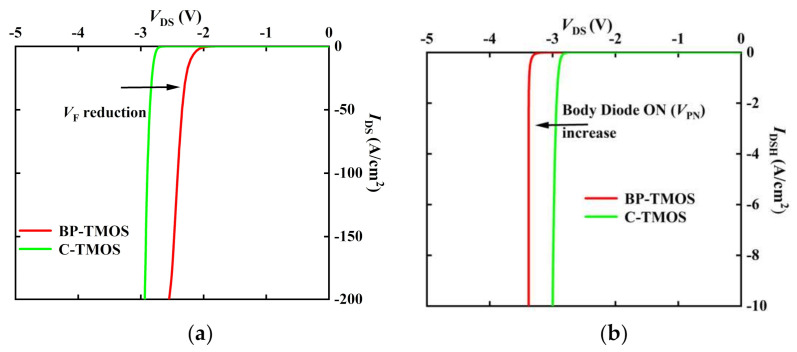
(**a**) *V*_F_ curves; (**b**) *V*_PN_ curves.

Figure 6 shows the third quadrant I-V curves for C-TMOS and BP-TMOS. It is obvious that the reverse voltage (*V*_F_) of BP-TMOS is far below C-TMOS because of HJD; as a result, BP-TMOS has a small dead-time loss. Moreover, just as Figure 6b shows, when the drain hole current *I*_dsH_ = 10 A/cm^2^, the reverse voltage (*V*_PN_) of the BP-TMOS increases to 3.4 V, while the *V*_PN_ of the C-TMOS is 2.7 V. This indicated that BP-TMOS has a large turn-on voltage relative to the body diode; thus, unwanted consumption can be controlled.

**Figure 7:**
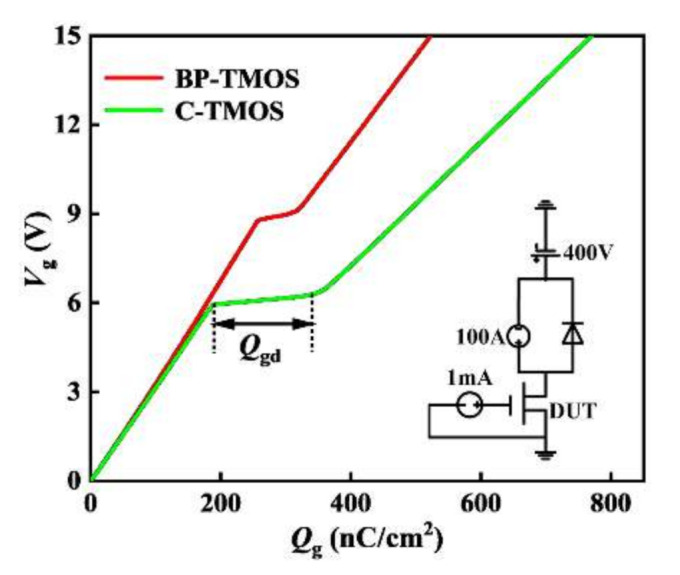
The dependences between the gate voltage (*V_g_*) and gate charge (*Q_g_*) of the devices. The extra insert is test circuit.

Figure 7 shows the dependences between the gate voltage (*V_g_*) and gate charge (*Q_g_*) of the devices. The active device area is set to be 1 cm^2^. The *Q_g_* and the gate-drain charge (*Q*_gd_) are 525 nC/cm^2^ and 50 nC/cm^2^ for BP-TMOS, respectively, which are both smaller than 775 nC/cm^2^ and 165 nC/cm^2^ of C-TMOS. This is because there is a L-shaped P+ layer in the BP-MOSFET, which reduces the overlap area between the drift region and the gate oxide.

**Figure 8:**
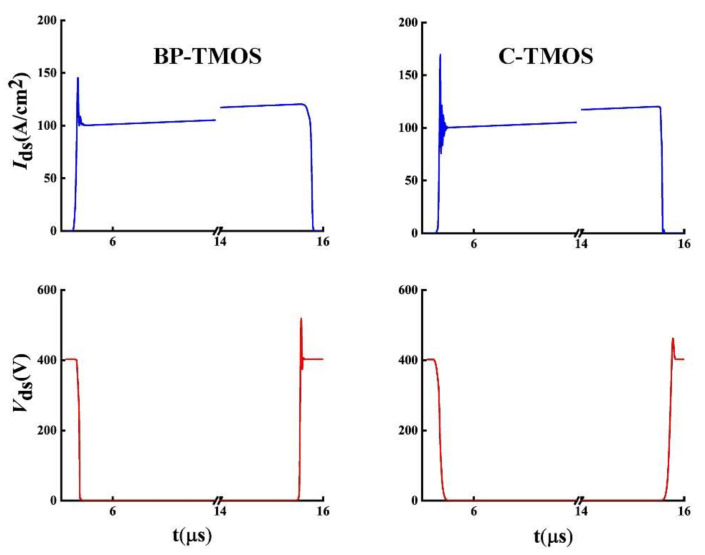
Switching waveforms for BP-TMOS and C-TMOS.

The switching performances analysis of the two devices used mixed-mode simulation in a double-pulse test. Figure 8 shows the switching performances for the two devices. Although SiC SBD has been used when simulating C-TMOS, BP-TMOS still has a small current spike in the turn-on state compared to C-TMOS.

**Figure 9:**
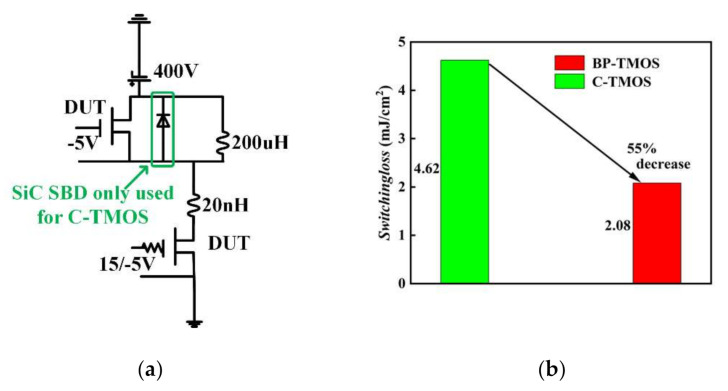
(**a**) Double pulse test circuit; (**b**) switching loss diagrams for the devices.

Figure 9 shows test circuit and switching losses. Figure 9b shows the switching loss results for the two devices. In comparison to C-TMOS, BP-TMOS has 55% decrease. The smaller *Q*_gd_ charge and the introduced heterojunction structure are the pivotal elements for the low switching loss. Table 2 summarises device performances, and BP-TMOS has a better comprehensive performances.

**Figure 10:**
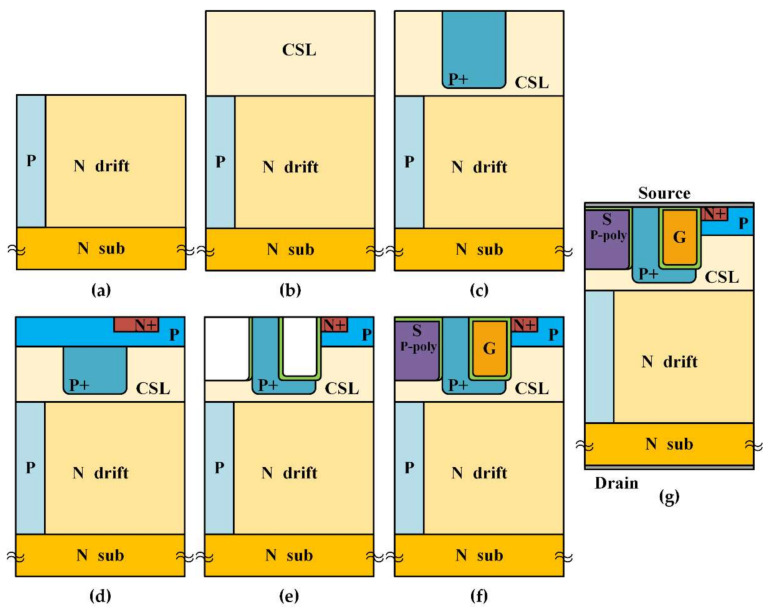
Process flow line of BP-TMOS. (**a**) N drift and P pillar grown; (**b**) CSL grown; (**c**) P+ shielding; (**d**) P channel and N + source regions; (**e**) Etch the gate trench and poly trench; (**f**) gate oxide and P-poly formed; (**g**) Metal electrodes.

A feasible fabrication procedure of the proposed device is provided in Figure 10. The double epitaxial layers including N drift and CSL are grown on the N substrate layer by chemical vapour deposition (CVD). The P-type layer in N drift used dose aluminium implantation. The P + shielding layer and the floating guard rings in termination region are formed simultaneously by heavy-dose aluminium implantation at 500 °C ambient temperature. After forming the P channel and N + source regions, all implants are activated at 1600 °C. The gate trench is formed by inductively coupled plasma (ICP) etching with a depth of 1.2 μm. After that, gate oxide and gate electrode are formed by thermal oxidation and low-pressure CVD processes, as well as P-poly in the dummy gate. Finally, the metal layers are deposited and sintered on the surface as well as the backside to form electrodes.

## 4. Conclusions

In this paper, a device was introduced which merged an HJD structure and a P-pillar in a SiC trench MOSFET. The merged HJD optimised the third quadrant performance. Near the gate trench, the electric field concentration can be reduced by the L-shaped high-doped P+ region. In addition, in the drift region, the buried region provides an assistant depletion effect. The breakdown voltage, the switching property, the reverse recovery characteristic, and the on-resistance are all promoted for the proposed structure.

## Figures and Tables

**Table 1:** The main parameters of the devices.

Parameter	C-TMOS	BP-TMOS	Unit
N drift doping, *N*_D_	7.5 × 10^15^	optimised	cm^−3^
N drift thickness, *L*_D_	8	7.6	μm
P+ layer doping, *N*_P_+	1 × 10^19^	1 × 10^19^	cm^−3^
P well doping, *N*_pwell_	3 × 10^17^	3 × 10^17^	cm^−3^
CSL layer thickness, *T*_CSL_	0.4	0.8	μm
CSL layer doping, *N*_CSL_	2 × 10^16^	2 × 10^16^	cm^−3^
P well thickness, *T*_pwell_	0.8	0.8	μm
Gate width, *W*_trench_	0.7	0.7	0.7
*L*_P-poly_	/	1	μm
*L*_P_	/	0.4	μm
P-pillar doping, *N*_P_	/	optimised	cm^−3^
P-pillar width, *W*_P_	/	optimised	μm
P-pillar length, *T*_P_	/	optimised	μm

**Table 2:** Basic device characteristics.

Parameter	C-TMOS	BP-TMOS
*R*_on,sp_ (mΩ·cm^2^)	1.86	1.48
*BV* (V)	773	1180
*Q*_g_ (nC/cm^2^)	775	525
*Q*_gd_ (nC/cm^2^)	165	50
*FoM* (MW/cm^2^)	321	940
*V*_F_ (V)	2.7	3.4

## Data Availability

Not applicable.
